# Strengthening the clinical laboratory workforce in Cambodia: a case study of a mixed-method in-service training program to improve laboratory quality management system oversight

**DOI:** 10.1186/s12960-020-00521-8

**Published:** 2020-11-04

**Authors:** Siew Kim Ong, Grant T. Donovan, Nayah Ndefru, Sophanna Song, Chhayheng Leang, Sophat Sek, Michael Noble, Lucy A. Perrone

**Affiliations:** 1 International Training and Education Center for Health (I-TECH) Cambodia, Phnom Penh, Cambodia; 2 Department of Global Health, Schools of Public Health and Medicine, International Training and Education Center for Health (I-TECH), University of Washington, Seattle, WA USA; 3 Department of Laboratory Medicine, School of Medicine, University of Washington, Seattle, WA USA; 4 Department of Pathology and Laboratory Medicine, University of British Columbia, Vancouver, BC Canada

**Keywords:** Laboratory quality management systems, Laboratory in-service training, Training methodology, Laboratory mentoring, Laboratory tele-mentoring

## Abstract

**Background:**

Laboratory diagnostic testing service delivery and compliance with international standards for laboratory quality are directly influenced by laboratory workforce competency. Many hospital laboratories in constrained resource settings such as Cambodia struggle to cope with the training needs of laboratory professionals in an environment of competing healthcare development priorities. Resource-limited countries need an adaptable and effective approach to provide laboratory professionals with job-specific quality oversight training to ensure the accuracy, timeliness, and reliability of diagnostic services.

**Case presentation:**

Here, we describe the results of an in-service training and mentoring program conducted with the Cambodia Ministry of Health at 12 tertiary-level hospital laboratories to drive improvements in laboratory quality management systems toward ISO 15189 accreditation, which demonstrated significant progress between baseline and outcome audits in a concurrent study. This case study describes the program, and evaluates how the four primary activities, including actionable gap assessments and planning, centralized and in situ training curriculum, in-person mentoring, and remote tele-mentoring via video communication technologies, contributed towards quality improvement in the participating laboratories. We evaluated participant responses to Likert scale and free response questions from program and training evaluation surveys, and we used thematic analysis to develop a model of best practices within the program’s four primary activities to inform future training approaches. Of these activities, participants agreed most highly that in-person visits and planning based on gap assessments contributed to their learning and ability to improve laboratory operations. Tele-mentoring was rated lowest by participants, who were critical of excessive group dialogue and distraction during web-conferencing; however, feedback suggests both in-person and remote mentoring contribute to continuing education, accountability to action, and peer collaboration and problem solving to improve workforce efforts toward improved quality management systems.

**Conclusions:**

We recommend here a package of in-service training activities for laboratory quality management system improvement initiatives in resource constrained settings that includes needs-based curricula and personalized action plans for participants; interactive and on-site training workshops; and in-person mentoring, complemented with well managed and regular tele-mentoring that focuses on knowledge retention, accountability to goals, and collaborative problem solving. Our model presents an adaptable approach to human resource development for quality improvement in medical laboratories.

## Background

Laboratory-based diagnostic testing plays a critical role in clinical decision-making and public health and accurate laboratory data are essential for informed decision-making [1–3]. Laboratory errors caused by poor quality management practices can lead to patient harm and a loss of trust by clinicians, resulting in the decreased use of diagnostic testing data for clinician decision-making and a cycle of poor quality, as often observed in resource-limited countries [4]. For the last 20 years, a significant international effort has been underway to assure a culture of quality and competence in laboratory testing through the implementation of laboratory quality management systems (LQMS) aligned with the International Organization for Standardization (ISO) ISO 15189 standard for medical laboratories [5]. This standard specifies requirements for quality and competence for medical laboratories globally; however, this standard is stringent and many countries lack the resources and trained personnel to achieve and maintain ISO 15189 accreditation without assistance [6]. Meeting national diagnostic services and international accreditation goals requires a laboratory workforce capable of complex organizational management and technical excellence. In low-resource settings, medical laboratory personnel are often limited by their educational and professional development opportunities and subsequent knowledge of the biological principles of diagnostic testing and quality assurance practices required to carry out testing procedures with repeatable accuracy [7].

In 2016, the first joint external evaluation was conducted to measure Cambodia’s achievements towards meeting the International Health Regulations. This revealed gaps in the areas of LQMS and workforce capacity, and additional investments in these areas were recommended to the Ministry of Health (MoH) [8]. The International Training and Education Center for Health (I-TECH) supported the Cambodia MoH Bureau of Medical Laboratory Services (BMLS) to implement a mentored LQMS strengthening program for 12 national and provincial hospital laboratories to improve quality management practices [9, 10]. The training and mentoring program presented in this case study began in July 2017 and included a package of practice-based in situ LQMS education and training activities, on-site mentorship, and frequent remote tele-mentoring support to all 12 laboratories. All technical assistance (TA) support was tailored towards improving each laboratory’s compliance with the ISO 15189 standard over the 2 years of implementation. Laboratory progress as a result of this TA was benchmarked against the CamLQMS assessment tool [a tool adapted from the WHO-AFRO Stepwise Laboratory Improvement Process Towards Accreditation (SLIPTA) tool and adopted in 2018 by the MoH as the primary assessment tool for laboratories in Cambodia]. The CamLQMS audits measured significant facility-level progress, with the 12 participating laboratories improving audit scores by an average percent difference of 21% (SD = 10%, Min = 7%, Max = 37%), demonstrating strengthened capacity of laboratory personnel to implement LQMS in their facilities [11]. A moderately strong correlation between audit performance and attendance time of laboratory personnel in remote mentoring activities suggested that remote mentoring could significantly contribute to substantial progress [11]; however, further examination of this correlation was warranted to better understand the impact of each element of the training and mentoring program and elucidate best practices for future programs. This examination is the subject of this case study.

## Case presentation

This training and mentoring program was implemented through four primary activities with the objective to educate and mentor staff in the 12 participating laboratories towards implementing a LQMS in compliance with the ISO 15189 standard. These included:

1. Design and implementation of laboratory audits and development of action plans with laboratories;
2. Development and implementation of a formal LQMS curriculum and practical application training in situ;
3. In situ mentoring of laboratory staff;
4. Remote training sessions and mentoring through video conferencing technologies.

### Design and implementation of laboratory audits and development of action plans with laboratories

In support of a MoH initiative to establish an improved national regulatory process over laboratory quality performance, this program provided training for 12 laboratory staff from participating laboratories to become professional LQMS auditors. This auditor training program was conducted over a period of 4 weeks via tele-conferencing and focused on LQMS principles, emphasizing ISO 15189 requirements (four power-point presentations with Khmer translation) and practice (each auditor trainee performed an audit independently using the CamLQMS checklist under the supervision of experienced auditors). The CamLQMS tool was utilized by the audit team to conduct baseline and exit audits of each laboratory participating in the program. The elements of the CamLQMS checklist specified national standards for LQMS conformity based on ISO 15189 and the Clinical and Laboratory Standard Institute (CLSI) QMS01-A4 guideline [12]. The checklist was divided into 12 sections organized by laboratory practice area and each section containing a series of quantifiable “yes”, “no” or “not applicable” questions to assess whether conformity was achieved. Numerical scores were derived for each section as well as overall performance. Laboratory audits were performed by teams of 4–5, consisting of members from I-TECH, the MoH-BMLS and at least one auditor trainee. Baseline audits were conducted in 2017 and identified areas of LQMS performance that were commonly low between the 12 participating laboratories. These results were discussed during a workshop with management and technical staff from the 12 laboratories in February 2018 and action plans developed. Program mentors trained participants to develop SMART (specific, measurable, achievable, realistic, and timely) [13] goals and action plans to address the gaps identified in the audits. Participants developed action plans collaboratively with peers, presenting implementation strategies and soliciting feedback from peer laboratories and quality improvement (QI) specialists. Following a 12-month period of laboratory-based QI work, staff training, and mentoring, audits were conducted again in 2019 to measure progress.

### Development and implementation of a formal LQMS curriculum and practical application training in situ

Information obtained during the audit process informed the development of a competency-based training curriculum for laboratory managers, quality assurance officers (QAOs), and other QI personnel in the laboratory, with training sessions implemented from resulting series of “off-site” and in situ workshops between February 2018 and April 2019. Four off-site workshops were conducted in Phnom Penh and were primarily focused on delivering LQMS and organizational leadership and management theory. These workshops used adult-learning strategies to engage participation and discussion, including dynamic simulation, experiential learning, peer collaboration, and transformative learning through problem solving. In situ sessions were designed for staff to apply LQMS theory using hands-on skills. Each training session included a combination of formal lectures by quality experts, peer discussion, case presentation and reflection, and experiential practice [14]. All training sessions were conducted in English and translated into Khmer by QI mentors or professional translators to ensure comprehension among non-English speaking participants. In addition, a limited-participant study tour to ISO 15189 accredited laboratories in Singapore was provided as an advanced learning opportunity for high performing laboratories, which motivated healthy competition among participants to complete action plan items to attend.

### In situ mentoring of laboratory staff

A team of four Cambodian QI mentors conducted regular site visits to laboratories for individual consultations with each visit designed to guide, support, and engage participants in QI efforts by:

- Providing one-on-one follow-up training with laboratories;
- Ensuring accountability of laboratory management and quality assurance personnel to action plans;
- Providing on-site technical guidance and collaboration to identify and resolve problems.

Mentors were dispatched a minimum of four times to each laboratory with laboratory specific TA and mentoring following workshops.

### Remote training sessions and mentoring through video conferencing technologies

The program’s tele-mentoring approach was modeled on successful clinical mentoring programs such as Project ECHO™ and used a similar “hub and spoke” structured format to advance knowledge and skills of laboratory staff [15]. Zoom (Zoom Video Communications, Inc., San Jose, California), a web-based video conferencing tool, was selected specifically for its low technical complexity, accessibility in low bandwidth settings, ability to connect multiple participants, video-recording and archiving features, and its screen sharing and remote desktop access functions. Mentors used Zoom to connect with laboratory managers and QAOs during weekly group trainings to reinforce formal training and to support ongoing action plans with troubleshooting assistance. Small group and individual meetings between mentors and participants were also conducted on an as needed basis. SMS communications were also utilized by QI mentors to connect with staff and answer questions in an ad hoc and individualized manner. Group SMS forums were established to advance group discussion between weekly Zoom sessions and enabled real-time *en masse* communication.

## Evaluation methodology

We used Likert scale survey responses to quantitatively assess participant ratings of the program’s primary activities on a scale of 1–5, then used qualitative observation and participant feedback to develop and present a model of best practices related to the program’s methodology, specifically identifying factors within the four primary program activities that likely facilitated or hindered the program’s success. Quantitative and qualitative data to assess best practices were collected primarily through a program evaluation survey, which assessed participant feedback after the program’s implementation with a combination of Likert scale and free response questions; these surveys were dispersed via email link to an online survey platform. The survey consisted of five statements regarding the program’s activities to improve LQMS systems in participating laboratories, which participants were prompted to rate their agreement with on a scale of 1–5 ranging from strongly disagree to strongly agree and explain their answers with written feedback. Participants consisted of laboratory managers, QAOs, and other quality assurance staff.

Additional qualitative data were collected from training specific, paper-based evaluations, which were carried out immediately after each individual training and expanded to include other training participants such as hospital and laboratory directors Post-training evaluations included open-ended prompts for feedback, which were recorded in a master spreadsheet by project staff, who translated comments from Khmer to English where necessary for qualitative analysis.

To analyze survey data, mean Likert scale ratings and response frequencies of each response were calculated to comparatively assess how well each program activity was perceived to contribute to QI within participant laboratories. Participant feedback was analyzed by a combination of inductive and deductive analysis. Prior to analysis of participant feedback, we compiled a hypothesized model of best practices within each of the primary program activities that contributed to program outcomes according to observation. We then analyzed feedback from surveys to corroborate and expand on this initial set of themes, categorizing text from participant comments into the relevant themes and inductively identifying new, previously unidentified themes recurring within the text. Using spreadsheets to organize and filter participant comments, each response was sorted according to the evaluation source and activity in question, then categorized into themes. Occurrence frequencies of each theme were tallied, analyzed, and then summarized.

## Results

Out of a total of 28 participants who were sent the program evaluation survey, 27 (96%) responded. Results demonstrated that participants agreed most highly that in-person mentoring and CamLQMS audits were valuable (means = 4.56 and 4.52, respectively), receiving the highest frequencies of “strongly agree” ratings and the highest mean ratings (Table 1). Conversely, participants agreed least with the statement that Zoom-based training and question/answer sessions were valuable to QI (mean = 3.44). Participants agreed moderately, however, with the statement that the overall program structure was effective in their laboratories’ improvement processes (mean = 4.11).

**Table 1:** Frequency distributions, total responses, and mean ratings of program evaluation survey responses

Survey category	Frequency (no.) of responses counted						
	1	2	3	4	5	Total responses	Mean rating
	Strongly disagree	Disagree	Neither agree nor disagree	Agree	Strongly agree		
CamLQMS action planning was valuable	0	0	0	13	14	27	4.52
Formal training was valuable	0	0	1	19	7	27	4.22
In-person mentoring was valuable	0	0	0	12	15	27	4.56
Zoom call check-ins were valuable	3	2	4	16	2	27	3.44
Overall program structure was effective	1	0	2	16	8	27	4.11

**Fig. 1:**
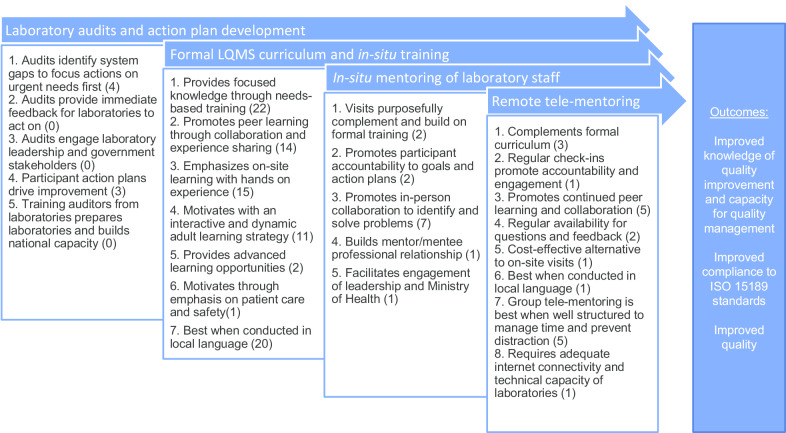
Model of best practice themes and the relationship of program activities to program outcomes. Numbers in parenthesis represent the number of participant comments that support each best practice theory.

Observations and qualitative analysis uncovered 23 themes regarding best practices, 21 of which were supported by participant feedback. We analyzed 239 individual comments from training evaluations and an additional 39 textual responses from program evaluation surveys. Responses from participants were brief, ranging from 1–68 words in length (median = 13). From these, we identified 147 instances of positive feedback and 99 instances of constructive critique for improvement, from which 123 comments either corroborated best practice themes or introduced novel themes according to the associated primary activity supported (Fig. 1).

### Best practices of laboratory assessments and action planning

Participant feedback indicated the “useful” role of the CamLQMS assessments in their QI progress, which provided a clear entry point for the continuous QI cycle, enabling participants to identify gaps and develop SMART action plans to address deficiencies. As one participant summarized: *“Plans* > *action* > *results* > *follow-up—Those process [sic] help to improve my lab quality”.* Although not corroborated by participant feedback, we also observed a clear benefit to participants from immediate feedback and corrective action by auditors during audits, and from auditors engaging the attention of both hospital leadership and government stakeholders.

The audit training program as well was observed to successfully engage selected laboratory staff in the audit process while supporting the MoH initiative to provide trained personnel for regulatory LQMS oversight and auditing. While a separate study would be required to compile and analyze feedback from these additional trainings, improved audit training is a recommended best practice to assure quality metrics.

### Best practices of in situ training

58 comments indicated improved learning due to practical training sessions, 22 of which referred to specific curriculum such as management review and equipment validation as being helpful. Additionally, peer learning and collaboration was remarked highly by participants, who often requested more peer learning activities where participants of different labs could share ideas and experiences, learning from each other and collaborating to troubleshoot and solve practical QI challenges. *“Every participant should raise, share, [sic] talk about experience*”, as one participant suggested.

Participants expressed considerable preference for in situ training activities, mentioning specifically the practical and experiential learning as important for participant improvement: *“This on-site lab training is so important, because we can practice directly and get new experience and skill”.* One of the key benefits to the in situ method was the advanced training and improvement opportunity that it gave to higher performing laboratories, which had the opportunity to present their own quality management systems for critical observation by their peer participants from other laboratories. Lower performing laboratories, meanwhile, were able to learn from the best practices of their peers. This practice addressed the need to match training content to benefit all ranges of participant experience. Participants were also able to acquire practical skills through direct hands-on participation during the experience. “*I think practice is better than theory*”, one participant suggested.

Finally, although our trainers strived to conduct all training in the local language wherever possible as a best practice, feedback from participants expressed strong critique for translators when information was not translated clearly enough. It was apparent that the training curriculum required further strengthening to meet the language needs of participants.

### Best practices of in situ mentoring

Participant feedback indicated strong value for face-to-face in situ mentoring and appreciated follow-up provided regarding QI plans and holding participants accountable. Participants appreciated the problem-solving suggestions of mentors, identifying and discussing problems on-site with participants and collaborating to overcome challenges to improvement. One participant commented: *“Mentor can see real situation in my lab and can suggest and help initiate my lab team to do something according to the gap.”* Participants also suggested that face-to-face visits from mentors served to improve the professional mentor–mentee working relationship, and further comments remarked that mentor visits had helped engage other important stakeholders such as hospital directors and MoH regulators in laboratory QI. One participant addressed both factors, stating “*Regular periodic visits from trainers is important because it makes an important connection between the training institute and the laboratory staff. An in-person trainer visit is also important to increase the credibility of the laboratory staff to the laboratory director.”* When mentors visit laboratories, MoH personnel and laboratory directors are invited to meet with mentors and QAO to discuss current and past improvement efforts, garnering support for laboratory improvement efforts; this engagement of leadership is critical.

### Best practices of tele-mentoring

A concurrent study of audit results found a significant correlation between participation time spent in Zoom tele-conferencing and laboratory QI [11]; however, participants in our study ranked Zoom tele-conferencing lower than all other program activities. Participants were critical of large group tele-conferencing activities in particular, stating “*too much talk with a lot of peoples [sic], difficult to take experience”,* “*talk for everything*”, and “*participants not [sic] pay attention during zoom call*.” This feedback suggests that improved facilitation structure and efficiency of video conferencing may be a best practice to improve the learning experience of participants, so long as the opportunity for peer learning is not sacrificed.

Another concern observed and heard from participants throughout the program, though surprisingly only addressed once through written feedback, is the challenge of unstable internet connections, which often decreased meeting productivity. In some locations, user desktop computers lacked a camera or webcam, which inhibited interactive participation. Assessing and improving technical capacity and internet connectivity should therefore be a necessary component of telementor program planning. Despite these challenges, participants noted improved knowledge from tele-mentoring sessions, appreciated regular feedback from mentors, and again praised the ability to collaborate with both peers and mentors, sharing experience and knowledge. Participants further praised the real-time access to QI mentors being able to ask questions and receive a response immediately, which was an important best practice of the mentors, who set regular open hours for participants to call for guidance. Training in the local language was once again a strong request from participants, and one comment noted the cost effectiveness of tele-mentoring, saving participants time and money required for travel. In addition to participant feedback, several strategies to address these challenges are recommended (Table 2).

**Table 2:** Recommended strategies to assure effective remote learning

Challenge	Recommended strategies for participants	Recommended strategies for facilitators
Peer learning environment resulted in significant chatter, distraction, ineffective time usage during remote learning exercises	Be familiar with and use other means to ask questions or communicate during sessions, such as using the chat box or hand raise functions to avoid interruptions Use headphones to avoid distracting feedback/echo Use a quiet area/room with minimal interruption from other routine work Avoid multitasking and assure attentiveness to avoid repeating questions or instructions	Do not assume competence; provide initial training on how to effectively use video conference platform Establish ground rules and group norms such as when questions are appropriate and how to use the chat box function Session weblink and agenda should be well planned and sent out ahead of time Invite participants to send in questions before the meeting starts, and have answers prepared Facilitator should make use of “mute-all” function when appropriate Designate a technical administrator to provide technical assistance during meeting Start meeting on time Send a summary of the learning points to participants after meetings Record training sessions and share previously recorded trainings with participants whenever possible Perform trainings in language of participants whenever possible; have a language translator available when needed Seek feedback from participants periodically to monitor and adjust practices
Unstable internet or electricity resulted in frequent dropped participants and interruption	Identify a stable internet connection Ensure that a back-up internet and/or power connection is available Use a mobile phone if it is a better option and does not incur extra costs on participants Test connectivity prior to meeting	Assess participant technical challenges prior to meeting and provide guidance or resources Provide list of common troubleshooting suggestions prior to meeting In cases of regular electrical shortages, check with national electricity supply board or monitor public notifications for potential outages
Lack of appropriate computer hardware or equipment such as a microphone and webcam reduces ability of attendees to participate	Laboratories should ensure access to a desktop computer with sound, microphone, and webcam Acquire and use quality headphones with built in microphone whenever possible Use smartphone (personal or institution-based) if necessary	Assess hardware capacity (audio-visual functionality) of participants Address access barriers: identify funding or resources for equipment, provide phone/data cards for participants

## Discussion

As demonstrated by measurable improvements in CamLQMS audit scores at the 12 laboratories, this training and mentoring approach made a positive impact in Cambodia and correlates with similar reports in the region [11, 16]. We found evidence that regular laboratory mentoring, supported by needs-based training and inter-laboratory collaboration enhances laboratory QI when it emphasizes actionable needs-based planning, peer learning in a practical and supportive environment, and collaborative problem solving. It was evident in the implementation of our program that customizing the mentoring and training program for the local environment is more effective than a preconceived one size fits all approach, and that taking an adaptive approach to teaching practice-based skills is critical for rapid QI [17].

Our program was designed specifically to utilize peer learning among a set of other dynamic, hands-on, and interactive adult-learning strategies implemented as adaptive drivers of change. Such strategies have been shown to be effective within healthcare professional training [14, 18], and participants gave a positive response to many of these training activities, for which we recommend them within our model of best practices. Our program further emphasized a focus on the interlinkage between quality and patient safety which motivated participants during training.

We recommend that tele-mentoring activities be designed to reinforce the continuous QI process, be results focused, encourage peer accountability, and collaborative learning and problem solving, and be conducted in the local language, wherever possible. We further recommend that mentors work with smaller groups or individual laboratories as much as possible for best results, maintaining the structure and control of the discussion, and establish time-bound question and answer periods to make the best use of participant time. Lastly, tele-conferencing success is linked to capable hosting and facilitation, reliable computing hardware with audio-visual capability and engaged participants. The importance of this capacity within health systems is visibly apparent during outbreaks of novel communicable diseases such as COVID-19, when physical distancing as a preventive measure hinders in-person training and communication [19]. The best tele-mentoring practices outlined in this case study may serve to inform academic programs transitioning to online platforms.

### Limitations

Because most of these best practice themes are derived first from implementing staff observations, this study may be influenced by positive biases toward the implemented activities and practices, missing potentially useful practices not observed by either staff or participants. Data from program participants too are limited by positive response bias from participants, and is further limited to written feedback only, which is limited to short answer responses. Extensive interviews of participants or key stakeholders may provide additional information. Program evaluation responses were further limited by language barriers among participants, who had varying abilities to respond clearly to the surveys in English, which resulted in some unclear or truncated responses. Feedback from training evaluations, which were responded to in either English or Khmer, contains the potential for translation error and bias as responses were translated into English by mentors rather than professional translators.

### Recommendations and conclusion

This study shows how a customizable package of needs-based planning, training, and mentoring can lead to improved laboratory QI via an informed, trained, and empowered workforce. Our model of best practices serves as a guide for future laboratory and health workforce development programs in Cambodia and globally. Participant feedback indicates the usefulness of in situ training and peer learning, and regular contact with experienced laboratory professionals who can provide both theoretical and practical coaching, hold participants accountable to goals, and enable collaboration to identify and solve problems. Support from local QI mentors and tele-mentoring was also associated with improved outcomes. We further recommend that in-person mentoring be maintained to some degree in such programs, due to its perceived value among mentors and participants in the improvement process. The successful blended learning package of activities including the auditor training program suggest that national scale-up would be a worthwhile investment in Cambodia. National entities responsible for health workforce development could model the successful elements of this program such as video conferencing for training and mentoring in order to reach more working professionals, engage more facilities in a national quality improvement effort, and utilize remote technology more fully as part of a staff competency assessment program and become a platform for delivering professional development opportunities in Cambodia.

## Data Availability

Any data generated in this program is available by request to the corresponding author.

## Abbreviations

BMLS: Bureau of Medical Laboratory Services
CamLQMS: Cambodian Laboratory Quality Management System checklist
CLSI: Clinical and Laboratory Standard Institute
ISO: International Organization for Standardization
I-TECH: International Training and Education Center for Health
LQMS: Laboratory quality management system
MoH: Ministry of Health
QAO: Quality Assurance Officer
QI: Quality improvement
QM: Quality management
QSE: Quality systems essentials
SLIPTA: Stepwise Laboratory Improvement Process Towards Accreditation
SMART: Specific, Measurable, Achievable, Relevant, and Time-bound
TA: Technical Assistance
WHO: World Health Organization
